# Effects of Nutrient-Fortified Milk-Based Formula on the Nutritional Status and Psychomotor Skills of Preschool Children

**DOI:** 10.1155/2017/6456738

**Published:** 2017-09-18

**Authors:** Mavil May C. Cervo, Diane S. Mendoza, Erniel B. Barrios, Leonora N. Panlasigui

**Affiliations:** ^1^School of Nutrition, Philippine Women's University, 1004 Manila, Philippines; ^2^College of Education, University of Santo Tomas, 1004 Manila, Philippines; ^3^School of Statistics, University of the Philippines Diliman, 1101 Quezon City, Philippines

## Abstract

This randomized, single-masked, controlled trial examined the effects of nutrient-fortified milk-based formula supplementation on nutritional status, nutrient intake, and psychomotor skills of selected preschool children with mean age of 4.10 ± 0.14 years. The study participants were divided equally into three major groups, normal, underweight, and severely underweight based on WHO-Child Growth Standards, and were further divided into two groups: fortified milk group who was given two glasses of fortified milk (50 g of powdered milk/serving) a day for twelve weeks in addition to their usual diet and the nonintervention group who was not given fortified milk and thus maintained their usual intake. Anthropometric measurements, dietary intake, and psychomotor developmental score were analyzed. Results showed that consumption of two servings of fortified milk a day for twelve weeks significantly increased the height of preschool children by 1.40 cm, weight by 1.35 kg, body mass index by 0.96 kg/m^2^, mid-upper arm circumference by 0.66 cm, and psychomotor scores by 13.74% more than those children who did not consume fortified milk (*p* < 0.0001). Hence, fortified milk-based supplement in the diet of preschool children improved overall nutritional status, nutrient intake, and performance in psychomotor scale. This study is registered in Philippine Health Research Registry: PHRR140923-000234.

## 1. Introduction

Childhood malnutrition remains a major problem in developing countries. In the Philippines, only marginal improvements in the nutritional status of children have been observed over the past decades. According to Food and Nutrition Research Institute of the Department of Science and Technology (FNRI-DOST), in 2013, almost 20% of Filipino preschool children are underweight and 30% are stunted. The prevalence of underweight and stunting increased with age while wasting is more pronounced among the younger age group (<2 years old) [1, 2]. About five million children showed signs of poor nutrition.

Consequences of malnutrition include a reduced resistance to infection resulting in infection being a leading cause of death among young children in developing countries [3–6]. Nutrition also plays a key role in the development of cognition and psychomotor skills since nutrients portray specific roles in supporting brain growth during fetal and early postnatal life. Iron, for example, is crucial for the production of neurotransmitters and important in energy metabolism; zinc in DNA synthesis and protein is essential in cell proliferation [7–9]. Numerous studies have demonstrated a correlation of growth indicators such as weight, height, and head circumference to children's intellectual abilities such that a poor nutritional status results in lower school performance, higher absenteeism, and a lower intelligence quotient [10–16]. The preschool period is a particularly sensitive period for the development of fundamental movement skills. Because most preschool children are naturally curious and love to play and explore, these fundamental movement skills are learned easily. The mastery of certain fundamental movement skills is a prerequisite for daily life functioning and participation in later physical or sport-specific activities [17].

The preschool period is a vulnerable stage for nutrient insufficiency, especially following the transition from breast feeding. Caloric requirements remain high and other nutrient rich foods may not be consumed due to food pickiness and/or a lack of food availability which can make the delivery of adequate nutrients needed to support growth and development challenging. Thus, providing energy and nutrient-dense food could be of great help by adding substantial amounts of nutrients to a child's regular diet.

The 7th Filipino National Nutrition Survey (2008) reported that consumption of fortified and unfortified milk and milk products was observed to provide an average of 38% of the mean one-day food intake of children aged 6 months to 5 years [1]. Milk is well-accepted by preschool children and fortified milk is therefore a good vehicle for the delivery of micronutrients, in particular iron and zinc [6, 18, 19]. Although the Filipino food pyramid recommends intake of at least one serving of dairy products per day in children 1–6 years of age, milk consumption remains inadequate in many poorer communities. Only 27.6% of children aged 6 months to 5 years drink milk [1]. Most Filipino children have low energy and nutrient intakes [20] which may have long term effects not only in their anthropometric measurements but also in their psychomotor abilities [10–16]. Identifying viable means of providing significant nutrients therefore may be of high importance. This study was designed to investigate the effects of a twelve-week community-based intervention that encouraged consumption of two servings of fortified milk in selected preschool children aged three to five years. Primary outcomes were growth measured as weight, height, body mass index, and mid-upper arm circumference. Nutrient intake was also determined using three-day food record and psychomotor skills were assessed to determine if the intervention improved performance.

## 2. Materials and Methods

### 2.1. Experimental Design

**Figure 1:**
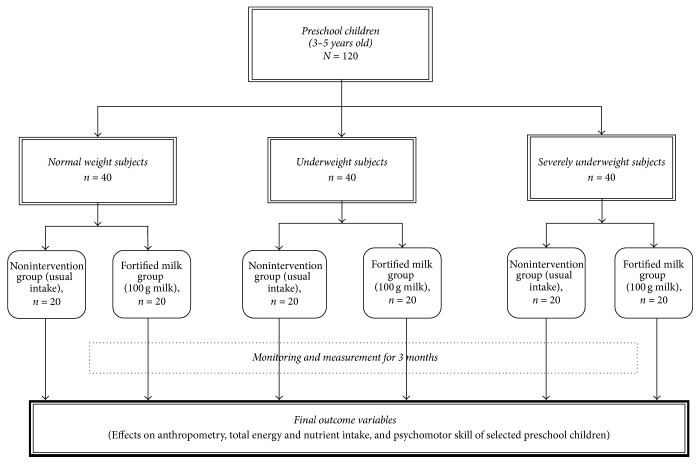
Experimental design (*n* = 120).

This randomized, single-masked, controlled study involved 120 subjects divided equally into three groups based on World Health Organization-Child Growth Standards (WHO-CGS) weight for age classification: normal, underweight, and severely underweight. The three groups were further randomly divided into two subgroups: those who were not given fortified milk and thus maintained their usual intake (nonintervention group) and those who were provided with 100 g of a chocolate and vanilla flavored nutrient-fortified milk-based formula (50 g powder Lactum® powder, Mead Johnson Nutrition, Inc., Makati City, Philippines, mixed in 200 ml water given twice daily) for twelve weeks (Figure 1).

### 2.2. Study Site

The study was conducted in Cardona, Rizal (Philippines), a town located in the southern part of Rizal province consisting mostly of hills and narrow plains. Its long shoreline facing the Laguna Lake provides the town with a thriving fishing industry, aside from agricultural lands and livestock. The town consists of 18 barangays. Prevalence of malnutrition at ages 0–5 years in this province is reported to be 20.5% [21].

### 2.3. Study Participants

One hundred twenty (120) preschool children, aged 3–5 years, with mean age of 4.10 ± 0.14 years were recruited for the study. Eligible subjects were divided into three groups, normal (NO), underweight (UW), and severely underweight (SU), and then randomized to either nonfortified milk group or fortified milk group using opaque sealed envelopes. Mean age for the fortified milk group (FMG) was 4.00 ± 0.13 years old and 4.30 ± 0.14 years old for the nonintervention group (NIG). Sixty-three percent (63%) of the 120 subjects were female and 33% males. Fifty-eight percent (58%) from the fortified milk group (FMG) were females and 42% are males, while 67% and 33% from the nonintervention group (NIG) are females and males, respectively.

Inclusion criteria were normal, underweight, and severely underweight children aged 3–5 years, with parents' or guardians' consent, no known allergy to milk and products, a permanent resident of the area, no severe illness, and no history of drinking milk for the past three months. Exclusion criteria were experiencing diarrhea, bloating, or pain in the abdomen following milk consumption previously, those with known lactose intolerance, overweight and obese children, a previous history of any vitamin or mineral supplements requirement for an appetite stimulant or any pharmacotherapy within the past 2 months, recent history of parasitism and chronic illness, or likelihood of a requirement for hospitalization or medications treatment for a chronic illness.

All parents or guardians of children aged 3–5 years from Cardona Rizal were given a pretested and validated survey form to assess whether their children fit the set inclusion criteria. The survey form was verified using a one-on-one interview with the parents. Those who met the selection criteria (195 preschool children) underwent anthropometric assessment (weight and height). Children who were classified as overweight and obese were excluded from the study. Potential participants were oriented with their parents or guardians regarding the details of the study. Parents or guardians of amenable subjects signed a volunteer consent form. The study followed the Declaration of Helsinki and ethical research guidelines by the Institutional Ethics Review Committee.

### 2.4. Test Food

The fortified milk provided to the intervention group meets the Filipino regulatory requirements for a powdered milk drink product and the Codex Standard for Follow-up Formula (Codex STAN 156-1987). The nutrient contents are detailed in Table 1.

### 2.5. Feeding Intervention

The administration of test food (fortified milk) lasted for twelve weeks. The nonintervention group (NIG) was not given milk thus maintaining their usual intake. Experimental group (fortified milk group) was instructed to consume 50 g of fortified milk vanilla flavor and 50 g of fortified milk chocolate flavor every day for twelve weeks. Each serving of the milk formula (50 g) was diluted in a 200 mL of distilled water. The fortified milk supplement was distributed to the intervention group on weekdays in Cardona Rizal's Municipal Social Welfare and Development Office (Philippines). For weekends, the parents were provided prepacked fortified milk formula powder and were instructed to give one packet in the morning and one in the afternoon, mixed appropriately with water. To ensure compliance, empty sachets were collected in the morning of the next weekday. Parents or guardians as well as the subjects were interviewed about consumption to further ensure compliance. Random spot checking of the households (ten households for each stratum or 30 household visits/day) was done. Participants' guardians were advised to contact any of the researchers if the participant was not able to consume the test formula due to illness or typhoons. The parents or guardians then received the test formula for the corresponding days the child was absent. Parents who failed to seek consent for their child's absence were visited in their houses and were given prepacked fortified milk formula powder. The nonintervention group was not asked to visit the study site except on the days when they underwent monitoring; thus there was a difference in frequency of contact with study personnel between the nonintervention and the intervention groups. After 12 weeks of intervention, the researchers still provided free milk samples to the study participants and rehabilitate the underweight and severely underweight children in the control group who were not given fortified milk during the intervention. The researchers also tie up with the town's Department of Social Welfare and Development as well as the town's Health Center to address the needs and ensure the welfare of the undernourished children.

The two servings of milk were based on the premise that it will lead to an increase in weight by approximately 1 pound per week. The study also aimed to determine if 2 servings of fortified milk are adequate enough to improve the nutritional status and psychomotor skills of undernourished children. The Filipino food pyramid for children (1–6 years old) recommends consuming 1 serving of milk a day yet there is still an increasing prevalence of macro- and micronutrient deficiency in the country.

### 2.6. Data Collection and Analysis

The subjects were monitored and assessed using different parameters. Socioeconomic and demographic profiles of the subjects and their families were gathered using a questionnaire before the study commenced. Anthropometric measurements were obtained every three weeks. A platform balance (Detecto Eye Level Physician Scale, MO, USA) was used to measure weight, microtoise (Tanita Corporation, Tokyo, Japan) for the height, and anthropometric tape for the mid-upper arm circumference of the subjects. The body mass index or BMI (kg/m^2^) was calculated based upon these measurements. Mid-upper arm circumference was classified according to Shakir and Morley's Classification system [22].

Weight was measured in the morning (before feeding time) and after the bladder has been emptied. Platform balance was placed on firm flooring. The subjects were instructed to remove his/her shoes and heavy clothing, such as sweaters or any pocket items, and was measured with both feet in the center of the scale. In measuring the height, the subjects were asked to remove their shoes and any hair ornaments and were advised not to put styling gel (especially among boys) that may interfere with taking measurements. Height measurement was taken on flooring that is not carpeted and against a flat surface such as a wall with no molding. Subjects were measured with feet flat, together, and against the wall, legs are straight, arms are at sides, and shoulders are level; head, shoulders, buttocks, and heels are touching the flat surface (wall). Researcher's eyes are at the same level as the headpiece. Mid-upper arm circumference was taken at midpoint of acromion process on shoulder blade and olecranon process of the ulna. Subjects were asked that forearm and palm are down across the body. Height, weight, and mid-upper arm circumference were measured three times to ensure reliability of results.

To estimate the energy and nutrient intake of the study participants, a three-day food record, two nonconsecutive weekdays and one weekend, was administered every month until the end of the study and was validated through one-on-one interview. The Philippine Food Composition Table (FNRI-DOST, 1997) and Food Exchange Lists for Meal Planning (FNRI-DOST, 1994) were used to quantify the dietary intake of the subjects [23]. Adequacy of the diet was assessed using Estimated Average Requirement (EAR) and Recommended Energy and Nutrient Intake (RENI) [24]. psychomotor development score was determined by child development specialists using a pretested developmental checklist based upon Dr. Simpson's Taxonomy of Psychomotor Domain [25]. Tests were performed before and after the intervention period. The developmental checklists include areas of (1) perception or the ability to use sensory cues to guide motor activity and ranges from sensory stimulation, through cue selection, to selection; (2) set or readiness to act, including mental, physical, and emotional sets which are dispositions that predetermine a person's response to different situations; (3) guided response or the early stages in learning a complex skill that includes limitation and trial and error, wherein adequacy of performance is achieved by practicing; (4) mechanism or the intermediate stage in learning a complex skill in which learned responses have become habitual and movements can be performed with some confidence and proficiency; (5) complex or overt response which is the skillful performance of motor acts that involve complex movement patterns; proficiency is indicated by a quick, accurate, and highly coordinated performance, requiring a minimum of energy, and includes performing without hesitation and automatic performance; (6) adaptation or skills that are well developed and the individual can modify movement patterns to fit special requirements; and (7) origination or creating new movement patterns to fit particular situation or specific problem; learning outcomes emphasize creativity based upon highly developed skills.

Compliance and potential adverse events were monitored using a daily diary provided to each subject's parents. The time the fortified milk was consumed in the afternoon was checked every day and during spot checking at other times. Adverse events monitored included diarrhea, abdominal distension, flatulence, nausea, or any potential allergic reactions.

All members of the research team have the necessary background and skills needed for the study. They were oriented and trained on the data collection techniques to ensure the uniformity of the methods that were employed. The child development specialists, those who conducted the anthropometric and dietary assessment, and the statistician were blinded to the group assignments of the study participants to prevent bias. All forms were pretested and validated prior to the actual study. Collected data were reviewed for completeness and accuracy by the principal investigators.

### 2.7. Statistical Analysis

This randomized, single-masked, controlled experiment involved 120 subjects divided into two major groups (nonintervention group and fortified milk group) and were further divided into three subgroups (normal weight, underweight, and severely underweight), with 20 subjects each. This number of sample size gave 88% statistical power at 5% significant level which is enough to detect significance since minimum power needed is 80% (16 subjects per stratum). Sample size was based from the expected 5% difference on weight and height before and after the intervention. Changes in different parameters were determined from baseline until the end of study intervention. All anthropometric measurements were performed in triplicate. Results were presented as mean ± standard error (SE). The standard errors of each mean for the anthropometric and dietary data were shown in order to give an idea of the dispersion of the mean. The test for significance of differences among the means of the anthropometric, energy and nutrient intake, and psychomotor score was analyzed using the analysis of covariance (ANCOVA) with baseline characteristics serving as the covariates. Differences across groups were further compared using Duncan Multiple Range Tests (DMRT). Analysis of covariance further included sex/gender as a covariate, and inherent differences between males and females in psychomotor function were accounted in the estimation of psychomotor outcome. Pearson's correlation coefficient was used to analyze any relationship between nutritional status and psychomotor scores. The SAS version 9.1 data analysis software (SAS Institute Inc., NC, USA) was used in testing for the significance among the data. Microsoft Office Excel 2010 (Microsoft Corporation, WA, USA) was used in processing other numerical data gathered.

## 3. Results

### 3.1. Baseline Characteristics of Subjects

One hundred twenty preschool children aged 3–5 years were included in this study. Mean age of the subjects is 4.10 ± 0.14 years old. Sixty-three percent of the subjects were female and 33% were males.  Table 2 shows the baseline characteristics of subjects.

### 3.2. Anthropometric Measurements

Table 3 shows the anthropometric measurements of subjects before and after the study intervention. After twelve weeks of supplementation, there was a significant impact of fortified milk supplementation on height (*p* < 0.0001), weight (*p* < 0.0001), body mass index (*p* < 0.0001), and mid-upper arm circumference (*p* < 0.0001). Nutritional status, baseline anthropometric measurements, sex, and age did not significantly affect these findings. Significant increases in growth parameters were observed in all groups, regardless of the baseline nutritional status. Adjusting for covariates, the fortified milk group had an average gain in height by 2.70 cm, weight by 1.66 kg, mid-upper arm circumference of 0.87 cm, and body mass index of 0.90 kg/m^2^. The nonintervention group's height, weight, and mid-upper arm circumference also augmented by 1.30 cm, 0.31 kg, and 0.21 cm, respectively, while the body mass index decreased by 0.06 kg/m^2^.

Among the fortified milk group, normal weight subjects had the highest increase in height (3.21 cm) and weight (1.97 kg), followed by the underweight (2.59 cm, 1.74 kg), and severely underweight group (2.29 cm, 1.26 kg). In terms of mid-upper arm circumference and body mass index, underweight subjects of fortified milk group had the highest increase (1.06 cm, 1.04 kg/m^2^), followed by normal weight (1.03 cm, 0.92 kg/m^2^) and severely underweight subjects (0.51 cm, 0.75 kg/m^2^), respectively.

Results indicate that consumption of 100 g (2 servings) of powdered fortified milk improved the height of children by 1.40 cm, weight by 1.35 kg, body mass index by 0.96 kg/m^2^, and mid-upper arm circumference by 0.66 cm more than those children who did not consume fortified milk.

Table 4 shows the classification of subjects based on different anthropometric indices as analyzed using WHO-Child Growth Standards (*z*-scores) after 12 weeks of study intervention. Although there was a significant increase in the weight of the subjects under the fortified milk group after twelve weeks of study intervention, only twenty (33%) of the subjects under the fortified milk group improved their nutritional status based on the WHO-CGS weight for age classification. Two of the subjects under the FMG-NO (fortified milk group with normal weight based from WHO-CGS weight for age classification) became at risk for possible overweight based on BMI for age. Upon examining the data, these subjects' baseline weight is in the upper limit of normal classification (borderline); thus the increase in weight after 12 weeks of intervention led to an increased risk for overweight, though in the lower limit only. One of the normal weight subjects under the nonintervention group became underweight and three became severely underweight after twelve weeks of intervention.

Since there are subjects whose weight, height, and BMI for age are in the borderline for different classifications, the researchers focused more on the significant changes in these anthropometric variables (in terms of magnitude) from baseline until 12 weeks of intervention to determine the effect of fortified milk consumption on nutritional status rather than on different anthropometric indices.

### 3.3. Energy and Nutrient Intake

Table 5 reveals the actual mean energy and nutrient intake of subjects before and after supplementation. Change in energy and nutrient intake after twelve weeks was significantly affected by milk supplementation at *p* < 0.0001. Covariates such as age, sex/gender, baseline nutrient intake, and nutritional status did not significantly affect the change in energy and nutrient intake of subjects after twelve weeks of study intervention. However, baseline anthropometric measurements significantly affect the final measurements; hence, the treatment effects were adjusted accordingly based on the covariance model. Dietary intake of subjects under the nonintervention group slightly decreased after twelve weeks of study. Energy and nutrient intake of fortified milk group subjects increased greatly after inclusion of milk in their diet. It is also evident that their dietary intake other than that provided by the fortified milk did not change substantially during the study. After twelve weeks, there is significant change in energy and nutrient intake in the fortified milk formula group compared with the nonintervention group. The fortified milk group increased their energy intake by 490 kcal, protein with 18.9 g, fat with 16.0 g, carbohydrates with 72.1 g, fiber with 3.4 g, calcium with 888 mg, iron with 7.96 mg, vitamin A with 367 ug RE, thiamin with 0.32 mg, riboflavin with 0.73 mg, niacin with 7.30 mg, and ascorbic acid with 36.56 mg. The nonintervention group slightly decreased their intake in terms of energy (73 kcal), protein (1.6 g), fat (2.0 g), carbohydrates (12.3 g), fiber (0.03 g), calcium (34 mg), iron (1.47 mg), vitamin A (11 ug RE), thiamin (0.20 mg), riboflavin (0.12 mg), niacin (1.05 mg), and ascorbic acid (5.69 mg). Net gain due to fortified milk supplementation following adjustment for covariates includes energy (563 kcal), protein (20.5 g), fat (18.0 g), carbohydrates (84.4 g), fiber (3.43 g), calcium (922 mg), iron (9.43 mg), vitamin A (378 ug RE), thiamin (0.52 mg), riboflavin (0.85 mg), niacin (8.35 mg), and ascorbic acid (42.35 mg).

The adequacy of nutrient intake as percent of Recommended Energy and Nutrient Intake (RENI) and Estimated Average Requirement (EAR) in the nonintervention group with normal weight group did not meet either the EAR or at least 80% of the RENI for energy, vitamin A, calcium, and ascorbic acid after twelve weeks of intervention. The subjects in the nonintervention group who were underweight did not meet the EAR in terms of energy, vitamin A, calcium, and ascorbic acid as well as at least 80% of RENI for energy, protein, vitamin A, calcium, and ascorbic acid. Mean intake of subjects in the nonintervention group who were severely underweight did not meet the EAR for energy, vitamin A, calcium, and ascorbic acid as well as 80% of RENI for energy, protein, iron, vitamin A, calcium, thiamin, and ascorbic acid. In contrast, all subgroups in the intervention group that received the fortified milk exceeded the EAR and RENI for all nutrients studied (Table 6).

Key nutrient intakes amounts in all groups were compared with the tolerable upper intake level (UL) suggested by FAO/WHO Recommended Nutrient Intakes Standard [26]. No subgroups in the nonintervention or the fortified milk group exceeded the UL for iron, vitamin A, calcium, or vitamin C. In the normal and underweight fortified milk supplemented group, niacin intake slightly exceeded recommended UL.

### 3.4. Psychomotor Skills

The psychomotor score improved more in the fortified milk group compared to the nonintervention group during the twelve weeks' study period (*p* < 0.0001). The nutritional status (*p* < 0.9246), sex (*p* < 0.6813), baseline psychomotor scores (*p* < 0.5465), and age as covariates (*p* < 0.9218) did not significantly affect the observed change in psychomotor score after twelve weeks. Changes in the psychomotor score after twelve weeks among nonintervention group and fortified milk group were* 5.27%* and* 19.01%*, respectively. This means that consumption of fortified milk-based formula for twelve weeks is associated with improvements in psychomotor skills of preschool children by* 13.74%* (*p* < 0.0001). The individual psychomotor assessment items were also analyzed, but the overall score alone is sufficient to conclude that milk supplementation is associated with improvement in the psychomotor skills of children.

Differences on psychomotor scores among fortified milk group were observed at baseline with *p* < 0.023 (Table 7). Based on Duncan statistical test, the underweight and normal subgroups had similar psychomotor scores while differences in scores between underweight and normal versus severely underweight at baseline were observed. After twelve weeks of fortified milk supplementation, similarities in psychomotor scores in all subgroups of fortified milk group were observed. This suggests that fortified milk supplementation may have more effect in improving the psychomotor scores of severely underweight preschool children compared to underweight and normal weight subjects. This may be due to the improvement in the children's anthropometric and dietary intake [27]. As previously discussed, brain development relies on adequate supply of essential nutrients. Additionally, the provision of these essential nutrients at right time is critical.

The study also investigated whether nutritional status is correlated with psychomotor development.  Table 8 shows that weight and mid-upper arm circumference (MUAC) have weak positive correlation while BMI has a very weak positive correlation and height with very weak negative correlation with psychomotor scores of subjects, though only weight and MUAC are found to be statistically significant. As parameters of nutritional status, this result suggests that nutritional status can indeed be correlated with psychomotor development of preschool children, although only a weak correlation was derived from the study.

Subjects who consumed fortified milk for twelve weeks significantly improved in terms of putting puzzles together, in matching outlines in concrete objects, comparing and contrasting objects, identifying sequential patterns, repeating nonsequential digits, sequencing three pictures, identifying functions of body parts, standing on tiptoe, balancing and walking, hopping on one foot, hopping backward with two feet, skipping, galloping, jumping from stationary position, and jumping over small objects compared with baseline. Subjects in the nonintervention group did not progress in terms of hopping backward with two feet, identifying sequential patterns, and sequencing three pictures compared with baseline (Table 9).

## 4. Discussion

This study demonstrates that providing an oral nutrition supplement of two servings of a fortified milk-based formula for twelve weeks is associated with improvements in some anthropometric measures and of psychomotor skills among 3–5-year-old children. Twelve weeks consumption of fortified milk group increased the height of preschool children by 1.40 ± 0.04 cm, weight by 1.35 ± 0.04 kg, body mass index of 0.96 ± 0.04 kg/m^2^, and mid-upper arm circumference of 0.66 ± 0.01 cm. Findings in the present study are consistent with studies on the effect of milk supplementation on anthropometric status of children [11, 16, 20]. The average increase in height of a preschool child is about 2-3 inches a year or around 0.17–0.25 inch (0.43–0.64 cm) a month, while the average weight gain is about 5-6 pounds per year or 0.19–0.23 kg per month [8]. Based on the results of the present study, it is evident that, compared with the standard or expected average gain in height and weight of a preschool child, all groups who consumed fortified milk-based formula were able to meet and even exceeded it while the nonintervention group was just within the expected increase in height and did not even meet the standard or average weight gain for a preschool child. The increase in weight in fortified milk group can possibly be attributed to the increase in muscle and bone mass of the children, as also cited in the study conducted by Albala et al. in 2008 [28], since the fortified milk used in this study is rich in nutrients like carbohydrates, protein, fats, calcium, phosphorus, magnesium, zinc, copper, and vitamins C, D, and K which are all needed in crystal and collagen formation, cartilage and bone metabolism, and even calcium and phosphate homeostasis, processes that are very significant during bone formation [29]. Since the growth in weight corresponds to a gain in height, risk of obesity is less as shown by the result of the body mass index for age. However, weight and height should be monitored to assess possible risk of obesity during longer period of supplementation. The study also showed that twelve-week fortified milk supplementation can induce significant weight gain, though it may not be enough to improve the nutritional status of the preschool children in terms of WHO-CGS weight for age classification so longer duration of intervention may be necessary. For a severely underweight 4.10-year-old child, there should be an increase of at least 1.50 kg weight to be classified as underweight and at least 1.6 kg to be classified as normal based on the WHO-CGS weight for age. In the present study, the average weight gain of the severely underweight group was 1.26 kg for the fortified milk group and 0.52 kg for the nonintervention group.

As observed in the study, the severely underweight group had the lowest gain in anthropometric measurements after supplementation of fortified milk. Although the severity of undernourishment would cause the body to adapt in such a way that nutrients are often absorbed more efficiently in order to compensate for the loss or to maintain homeostasis, there are also some instances where severe malnutrition can lead to intestinal changes such as atrophy which could contribute to a lesser nutrient uptake or absorption.

In terms of dietary intake, both groups have the same consumption at baseline. However at the end of the study, there was a significant increase in the nutrient intake of the fortified milk group (*p* < 0.0001). Increase in energy and nutrient intake was greatly influenced by the milk supplementation (*p* < 0.0001). It is apparent that energy and nutrient intake of the fortified milk group increased after twelve weeks of fortified milk inclusion in their usual diet without a decrease in the nutrient consumed from other foods (minus the fortified milk for experimental or fortified milk group) since the diet did not vary much from each other since the diet did not vary much from each other and every monitoring is somewhat monotonous since it was observed that the participants usually consume the same kind of foods almost every day. These findings are concurrent with the study of Huynh et al. (2016) which showed improvement on dietary intake of nutrients through oral nutritional supplementation (ONS). Provision of supplementation may provide significant amounts of nutrients and energy, which may contribute to dietary adequacy. Mean energy and nutrient intake of those in the nonintervention group slightly decreased after twelve weeks of intervention, though not statistically significant (*p* < 0.494).

Milk is rich in various nutrients. The powdered fortified milk-based formula given to the subject is a source of additional nutrients including energy, protein, fat, carbohydrates, vitamin A, thiamin, riboflavin, niacin, vitamins B_6_, B_12_, C, D, E, and K, folic acid, pantothenic acid, biotin, calcium, phosphorus, chloride, iodine, iron, zinc, manganese, copper, choline, dietary fiber, and linoleic acid as well as DHA or docosahexaenoic acid, which are all important in the growth and development of a child. However, in this study, we focused only on the nutrients that are present in the Philippine Food Composition Table as well as on the 2002 Recommended Energy and Nutrient Intake Table for the analysis.

A recommendation/requirement is an intake level which will meet specified criteria of adequacy, preventing risk of deficit or excess [24]. Based on the results of the study, all groups did not meet the RENI for energy, protein, vitamin A, and calcium before the study commenced. But fortified milk group eventually surpassed the requirement for each nutrient after supplementation of fortified milk. Most of the groups were able to meet the requirement for the B vitamins (thiamin, riboflavin, and niacin) since most of the subjects consumed rice and bread daily which are good sources of these vitamins. Also, for fortified milk group, the test food (fortified milk) is also a good source of B vitamins. B vitamins are important coenzymes in energy metabolism thus helping to provide the brain's energy supply for it to function very well. Vitamin B_1_ or thiamin, particularly, is important for brain development and function [30]. Children who were fed with thiamin deficient formula during infancy showed impaired language ability which proves the role of thiamin on brain development [31]. On the other hand, the nonintervention group's intake did not improve much after twelve weeks of study that it even increases its deviation from the RENI. Also, mean dietary intake of the group did not meet at least 80% of the RENI for energy, vitamin A, calcium, and ascorbic acid.

EAR (Estimated Average Requirement) is the average amount of a nutrient needed by a group of individuals in whom a functional or clinical assessment has been conducted and measures of adequacy have been made at a specified level of dietary intake [24]. The values for EAR of all nutrients were based on the FAO/WHO Recommended Nutrient Intakes Standard. In terms of EAR, all groups, both fortified milk group and nonintervention group, were not able to meet the average requirement for energy, vitamin A, ascorbic acid, and calcium at baseline. However, the fortified milk group showed a remarkable improvement of meeting all the requirements upon inclusion of fortified milk in the subjects' intake. In contrast, the nonintervention group showed no progress and even declined during the final phase of the study. Mean dietary intake of the nonintervention group did not meet the average requirement for energy, vitamin A, calcium, and ascorbic acid after twelve weeks of study.

At the stage where feeding difficulty with children are present together with increased needs for growth and development, ensuring complete nutrition becomes a challenge for all mothers, and the present study showed that milk supplementation could be one of the interventions given to address this problem. In the 2008 Philippine National Nutrition Survey, it was reported that milk consumption dramatically decreased as children grows, with 378 g whole milk during 6–11 months to 354 g during 12 to 35 months and 64 g during 36–71 months. As trend in milk consumption decreased, overall energy and nutrient intake of children of different age groups also declined, especially in terms of vitamin A, calcium, riboflavin, and ascorbic acid. Only 23.6% of children 6 months to 5 years and 2.8% of 6–12-year-old children met the EAR for calcium. Based on the 2008 NNS results, milk and milk products contribute 14% of 6 months to 5-year-old children's total energy intake, 19% protein, 56% calcium, 34% vitamin A, 33% vitamin C, 18% thiamin, 48% riboflavin, 30% niacin, and 20% iron [1]. Results of present study are consistent with the findings of the 2008 NNS that milk provides a significant amount of nutrients in children's diet and greatly helps in achieving the recommended energy and nutrient requirement.

The mean intake of the fortified milk group and nonintervention group was compared with the tolerable upper intake level (UL) of each nutrient for both 1–3- and 4–8-year-olds based on FAO/WHO Recommended Nutrient Intakes Standard. All subgroups under the fortified milk group exceeded the RENI but not the UL for all nutrients studied except for niacin, yet there is no fear for possible toxicity. Though the mean intake of the groups slightly surpassed the UL for niacin, this level has minimal if any risk, since niacin cannot be stored to any significant extent in the body and excess is excreted in the urine. Likewise, there is no evidence of adverse effects of an excess intake of naturally occurring niacin in foods and only those that are in the form of drugs (in very high dosage) can cause liver toxicity [8].

Physical and motor skills in early childhood involve three basic types of movements: (a) locomotor abilities such as jumping, running, hopping, skipping, and galloping, which relies on energy and also calcium for strong bones; (b) nonlocomotor abilities (balancing or stabilizing) such as turning, pulling, and other balancing activities; and (c) manipulative abilities (bouncing, kicking, and grasping) that include the operations and control of limited and precise movements of the small muscles, especially those in the hand and feet. Enough supply of energy and nutrients helps in ensuring proper development. Environment including the culture they have and even their livelihood, how open they are to situations they can socialize, and opportunities in which they practice motor skills are also factors that could affect psychomotor skills of children [32]. In this study, parents or guardians of the study participants were oriented and constantly reminded to not change anything (diet, physical activity, parenting style/climate, etc.) to ensure that no other factors may affect the results of the study. However, the researchers failed to measure or analyze in both groups if there was a change in family and parenting climate during the intervention, which is one of the limitations of our study.

Malnutrition can adversely affect brain development and performance especially during the early years of life [4, 33, 34]. Previous studies have investigated the effects of macro- and micronutrient deficiencies on cognitive and psychomotor development. Protein-energy malnutrition reduces IQ levels, cognitive function, and school achievement and is associated with greater behavioral problems [12, 35–38]. Iron deficiency alone is associated with reductions in cognition and psychomotor skills [39]. Idjradinata and Pollitt reported improvement in children's mental and motor scores after iron supplementation [40]. Iron and zinc supplementation has been shown to improve infant motor development [9] while zinc supplementation alone demonstrated superior neuropsychological performance, particularly reasoning, when compared with controls [41–43]. Children with higher copper and ferritin levels show better visual motor integration suggesting that deficiency has adverse consequences [33]. Long chain polyunsaturated fatty acids support synaptogenesis and myelin synthesis and deficiency during early development can impact long term cognitive performance [44].

An increase in psychomotor score was observed after the twelve-week intervention and those under the fortified milk group showed greater improvement. Moreover, fortified milk supplementation may have more effect in improving the psychomotor scores of severely underweight preschool children compared to underweight and normal weight subjects. The fortified milk group had higher percent change in their scores on comparing and contrasting objects, identifying sequential pattern, repeating nonsequential digits, and sequencing pictures. These cognitive-intellectual skills are important for their reading readiness by distinguishing the qualities of objects and eventually letters and words. Putting puzzles together suggests improved problem solving skills and/or better motor dexterity. There was also improvement in the fortified milk group's motor skills score that may be related to an improved ability to balance on one leg, stand on tiptoe and hop on one foot forward and backward, skip, gallop, and jump over small objects, and move vigorously compared with the nonintervention group which could be due to improved coordination and/or strength.

As discussed earlier, nutrition plays an important role in brain development and psychomotor performance. The improvement of nutritional status has a positive correlation with the development of psychomotor skills observed through a cross-sectional study of children 24–35-month-old as measured by weight for age, height for age, MUAC and head circumference, and psychomotor functioning [10]. This correlation was also observed in the current study, although a weak positive correlation exists between anthropometric measures such as weight and mid-upper arm circumference and psychomotor scores. Children who may have missed the critical window of opportunity for brain development may exhibit lower cognitive and psychomotor skills [13]. Though it is a limitation in the study, the extent of missed cognition and psychomotor skills may require a more individualized approach which may also include activities that may stimulate brain development alongside nutrition [27]. Possibly, a longer duration of supplementation may also be looked into.

Protein and energy are two of the major requirements in brain development because of its role in cell proliferation, cell differentiation, and growth factor synthesis, together with other micronutrients such as iron for myelin and monoamine synthesis and neuronal and glial energy metabolism; zinc for DNA synthesis and neurotransmitter release; copper for neurotransmitter synthesis and neuronal and glial energy metabolism and antioxidant property; long chain polyunsaturated fatty acids for synaptogenesis and myelin synthesis; and lastly choline for neurotransmitter synthesis, DNA methylation, and myelin synthesis. In this study, the fortified milk group was given an additional 433 kcal/day (15% RENI) and 15 g protein (20% RENI) from milk supplementation. The fortified milk also provided an additional 7 mg iron (43% RENI), 5.1 mg zinc (48% RENI), 350 mcg (40% DRI) copper, 40 mg choline (8% DRI), 7.8 mg DHA, and 180 mg linoleic acid from the 100 g/day milk supplementation. With additional nutrients essential for brain development coming from the milk supplemented to the fortified milk group, it is plausible that the increase in the psychomotor scores was mainly due to the milk supplementation (*p* < 0.0001) in the present study.

Future studies with a longer intervention period may lead to even more significant improvement and could provide guidance regarding the risk of obesity with prolonged supplementation. Biochemical parameters may also be included as measures of nutritional status. Family and parenting climate and other possible environmental factors should also be considered and analyzed. Test-retest reliability should also be done especially for psychomotor skills analysis.

## 5. Conclusion

Supervised consumption of two servings (100 g) of nutrient-fortified milk-based formula a day for twelve weeks improves the height, weight, body mass index, mid-upper arm circumference, and psychomotor skills of preschool children. Energy and nutrient intake of the fortified milk group increased and was able to meet the Recommended Energy and Nutrient Intake (RENI) and Estimated Average Requirement (EAR) for all important nutrients particularly energy, protein, calcium, iron, vitamin A, thiamin, riboflavin, niacin, and vitamin C of children 4–6 years old. Thus, inclusion of fortified milk-based formula in the diet of preschool children may not only help in the improvement of overall nutritional status but also help in the psychomotor skills of children which are vital in achieving their developmental milestones.

## Figures and Tables

**Table 1:** Nutrient content of test food (nutrient fortified milk based formula).

Nutrients	Nutrient fortified milk-based formula per 50 g, 1 serving (vanilla)	Nutrient fortified milk-based formula per 50 g, 1 serving (choco)
Energy, kcal	216	217
Fat, g	7	7
Linoleic acid, mg	90	90
Protein, g	7.5	7.5
Carbohydrates, g	32	32
Minerals (ash), g	2.2	2.2
Iron, mg	3.9	3.9
Vitamin A, IU	590	590
Vitamin D, IU	170	165
Vitamin E, IU	1.9	1.9
Vitamin K, IU	5.5	5.5
Vitamin B_6_, mg	270	270
Vitamin B_12_, mg	0.46	0.44
Folic acid, mcg	14	14
Calcium, mg	440	440
Thiamin, mg	0.19	0.19
Riboflavin, mg	0.36	0.36
Niacin, mg	3.5	3.5
Ascorbic acid, mg	19.5	20
Pantothenic acid, mcg	1050	1050
Biotin, mcg	6	6.2
Phosphorus, mg	230	230
Magnesium, mg	28	28
Sodium, mg	82.5	67
Potassium, mg	300	290
Chloride, mg	165	165
Iodine, mcg	36	36
Zinc, mg	2.6	2.6
Manganese, mcg	270	270
Copper, mcg	150	175
Choline, mcg	20	20
DHA, mg	3.9	3.9
Dietary fiber, g	1.5	1.5

**Table 2:** Baseline characteristics of subjects.

Variables	NIG-NO		NIG-UW		NIG-SU		FMG-NO		FMG-UW		FMG-SU	
	Mean	SEM	Mean	SEM	Mean	SEM	Mean	SEM	Mean	SEM	Mean	SEM
Age, years	48.0	3.13	48.0	3.13	48.0	3.13	51.0	3.54	53.0	3.83	52.0	3.61
Height, cm	100.53	1.38	98.33	1.27	94.45	1.28	99.17	1.40	95.08	1.01	91.85	1.34
Weight, kg	14.35	0.44	12.79	0.33	11.33	0.28	14.76	0.41	12.49	0.23	10.77	0.30
MUAC, cm	16.06	0.30	14.91	0.21	14.41	0.23	16.49	0.22	14.96	0.22	14.55	0.23
BMI, kg/m^2^	14.15	0.17	13.24	0.19	12.84	0.22	15.02	0.25	13.95	0.22	12.95	0.19
*Weight for age*												
*Normal (0 to −2 SD)*	*n* = 20						*n* = 20					
*Underweight (<−2 to -3 SD)*			*n* = 20						*n* = 20			
*Severely underweight (<−3 SD)*					*n* = 20						*n* = 20	
*Dietary intake*												
Energy, kcal	1258	63	1049	51	998	58	1164	55	1069	56	1025	55
Protein, g	33.1	2.1	27.7	1.2	27.7	2.1	30.1	1.7	3.4	1.8	29.5	2.0
Iron, mg	10.83	1.46	7.85	0.94	7.77	1.57	11.77	2.62	8.71	1.01	6.93	0.59
Vitamin A, mcg RE	131	15	110	12	101	11	120	15	120	12	109	9
Calcium, mg	253	24	197	14	210	24	253	25	231	25	213	13
Thiamin, mg	1.27	0.24	0.95	0.17	0.89	0.22	1.43	0.44	0.90	0.18	0.69	0.10
Riboflavin, mg	0.88	0.14	0.6	0.10	0.58	0.14	0.93	0.26	0.68	0.09	0.57	0.08
Niacin, mg	10.62	1.27	8.46	0.80	7.81	1.31	11.59	2.33	9.16	0.87	7.97	0.64
Ascorbic acid, mg	31	6	19	4	17	7	36	9	24	4	34	6

*Notes*. NIG-NO: nonintervention group with normal weight based on WHO-CGS weight for age; NIG-UW: nonintervention group who are underweight based on WHO-CGS weight for age; NIG-SU: nonintervention group who are severely underweight based on WHO-CGS weight for age; FMG-NO: fortified milk group with normal weight based on WHO-CGS weight for age; FMG-UW: fortified milk group who are underweight based on WHO-CGS weight for age; FMG-SU: fortified milk group who are severely underweight based on WHO-CGS weight for age.

**Table 3:** Change in anthropometric measurements among subjects after twelve weeks of intervention.

Group	Height, cm		Weight, kg		Mid upper arm circumference, cm		Body mass index, kg/m^2^	
	Baseline	Posttest	Baseline	Posttest	Baseline	Posttest	Baseline	Posttest
NIG-NO	100.53 ± 1.38	101.75 ± 1.32	14.35 ± 0.44	14.61 ± 0.48	16.06 ± 0.30	16.24 ± 0.30	14.15 ± 0.17	14.01 ± 0.19
NIG-UW	98.33 ± 1.27	99.64 ± 1.23	12.79 ± 0.33	12.94 ± 0.32	14.91 ± 0.21	15.04 ± 0.17	13.24 ± 0.19	13.04 ± 0.23
NIG-SU	94.45 ± 1.28	95.82 ± 1.25	11.33 ± 0.28	11.85 ± 0.28	14.41 ± 0.23	14.72 ± 0.16	12.84 ± 0.22	13.00 ± 0.17
FMG-NO	99.17 ± 1.40	102.38 ± 1.41	14.76 ± 0.41	16.73 ± 0.38	16.49 ± 0.22	17.52 ± 0.24	15.02 ± 0.25	15.94 ± 0.27
FMG-UW	95.08 ± 1.01	97.67 ± 1.04	12.49 ± 0.23	14.23 ± 0.27	14.96 ± 0.22	16.02 ± 0.20	13.95 ± 0.22	14.99 ± 0.26
FMG-SU	91.85 ± 1.34	94.14 ± 1.22	10.77 ± 0.30	12.03 ± 0.31	14.55 ± 0.23	15.06 ± 0.23	12.95 ± 0.19	13.70 ± 0.20

*Notes*. Computed as mean ± SE. All are significant at *p* < 0.0001.

**Table 4:** Classification of subjects based on different anthropometric indices as analyzed using WHO-Child Growth Standards (*z*-scores) after 12 weeks of study intervention.

Group	Weight for age classification		Height for age classification		Body mass index for age classification	
	Baseline	Final	Baseline	Final	Baseline	Final
NIG-NO	Normal: 100% (20)	Normal: 95% (19)	Normal: 100% (20)	Normal: 100% (20)	Normal: 90% (18)	Normal: 85% (17)
		Underweight: 5% (1)			Possible risk of overweight: 10% (2)	Possible risk of overweight: 15% (3)

NIG-UW	Underweight: 100% (20)	Underweight: 85% (17)	Normal: 85% (17)	Normal: 75% (15)	Normal: 85% (17)	Normal: 80% (16)
		Severely underweight: 15% (3)	Stunted: 15% (3)	Stunted: 25% (5)	Wasted: 15% (3)	Wasted: 15% (4)

NIG-SU	Severely underweight: 100% (20)	Underweight: 20% (4)	Normal: 30% (6)	Normal: 30% (6)	Normal: 30% (6)	Normal: 40% (8)
		Severely underweight: 80% (16)	Stunted: 45% (9)	Stunted: 45% (9)	Wasted: 55% (11)	Wasted: 45% (9)
			Severely stunted: 25% (5)	Severely stunted: 25% (5)	Severely wasted: 15% (3)	Severely wasted: 15% (3)

FMG-NO	Normal: 100% (20)	Normal: 85% (17)	Normal: 100% (20)	Normal: 100% (20)	Normal: 95% (19)	Normal: 85% (17)
		Possible risk of overweight: 15% (3)			Possible risk of overweight: 5% (1)	Possible risk of overweight: 15% (3)

FMG-UW	Underweight: 100% (20)	Normal: 45% (9)	Normal: 65% (13)	Normal: 75% (15)	Normal: 75% (15)	Normal: 85% (17)
		Underweight: 55% (11)	Stunted: 30% (6)	Stunted: 25% (5)	Wasted: 25% (5)	Wasted: 15% (3)
			Severely stunted: 5% (1)			

FMG-SU	Severely underweight: 100% (20)	Underweight: 40% (8)	Normal: 25% (5)	Normal: 35% (7)	Normal: 25% (5)	Normal: 40% (8)
		Severely underweight: 60% (12)	Stunted: 60% (12)	Stunted: 60% (12)	Wasted: 60% (12)	Wasted: 55% (11)

*Notes*. Weight for age: normal (0 to −2 SD); underweight (<−2 to −3 SD); severely underweight (<−3 SD); possible risk of overweight (>0 SD); height for age: normal (≤2 to −2 SD); stunted (<−2 to −3 SD); severely stunted (<−3 SD); BMI for age: normal (0 to −2 SD); wasted (<−2 to −3 SD); severely wasted (<−3 SD); possible risk of overweight (2 to >0 SD).

**Table 5:** Mean energy and nutrient intake of subjects before and after twelve weeks of intervention.

Nutrients	Nonintervention group (NIG)						Fortified milk group (FMG)								
	Normal weight (NO)		Underweight (UW)		Severely underweight (SU)		Normal weight (NO)			Underweight (UW)			Severely underweight (SU)		
	Baseline	Posttest	Baseline	Posttest	Baseline	Posttest	Baseline	Posttest (w/o fortified milk)^*∗*^	Posttest (w/fortified milk)^*∗*^	Baseline	Posttest (w/o fortified milk)^*∗*^	Posttest (w/fortified milk)^*∗*^	Baseline	Posttest (w/o fortified milk)^*∗*^	Posttest (w/fortified milk)^*∗*^
Energy, kcal	1258 ± 63	1114 ± 81	1049 ± 51	952 ± 51	998 ± 58	989 ± 56	1164 ± 55	1170 ± 55	1603 ± 44	1069 ± 56	1146 ± 55	1579 ± 51	1025 ± 55	1121 ± 55	1554 ± 54
Protein, g	33.1 ± 2.1	31.2 ± 2.6	27.7 ± 1.2	25.8 ± 1.1	27.7 ± 2.1	26.7 ± 1.7	30.1 ± 1.7	31.2 ± 1.7	46.2 ± 1.5	3.4 ± 1.8	33.0 ± 1.8	48.0 ± 1.8	29.5 ± 2.0	31.4 ± 2.0	46.4 ± 2.0
Iron, mg	10.83 ± 1.46	7.75 ± 0.92	7.85 ± 0.94	7.66 ± 0.94	7.77 ± 1.57	6.63 ± 0.65	11.77 ± 2.62	11.62 ± 2.62	19.42 ± 2.59	8.71 ± 1.01	9.07 ± 1.01	16.87 ± 1.00	6.93 ± 0.59	7.21 ± 0.59	15.01 ± 0.60
Vitamin A, mcg RE	131 ± 15	114 ± 12	110 ± 12	104 ± 11	101 ± 11	93 ± 8	120 ± 15	129 ± 15	487 ± 19	120 ± 12	132 ± 12	490 ± 12	109 ± 9	117 ± 9	475 ± 9
Calcium, mg	253 ± 24	198 ± 17	197 ± 14	185 ± 12	210 ± 24	175 ± 11	253 ± 25	258 ± 25	1138 ± 25	231 ± 25	241 ± 25	1121 ± 24	213 ± 13	221 ± 14	1101 ± 14
Thiamin, mg	1.27 ± 0.24	0.85 ± 0.12	0.95 ± 0.17	0.92 ± 0.18	0.89 ± 0.22	0.76 ± 0.14	1.43 ± 0.44	1.00 ± 0.44	1.38 ± 0.44	0.90 ± 0.18	1.00 ± 0.19	1.38 ± 0.19	0.69 ± 0.10	1.00 ± 0.10	1.38 ± 0.10
Riboflavin, mg	0.88 ± 0.14	0.58 ± 0.08	0.6 ± 0.10	0.61 ± 0.10	0.58 ± 0.14	0.49 ± 0.07	0.93 ± 0.26	0.92 ± 0.25	1.64 ± 0.25	0.68 ± 0.09	0.71 ± 0.09	1.43 ± 0.09	0.57 ± 0.08	0.57 ± 0.08	1.29 ± 0.08
Niacin, mg	10.62 ± 1.27	8.53 ± 0.90	8.46 ± 0.80	8.19 ± 0.90	7.81 ± 1.31	7.00 ± 0.67	11.59 ± 2.33	11.70 ± 2.28	18.70 ± 2.25	9.16 ± 0.87	9.66 ± 0.88	16.66 ± 0.88	7.97 ± 0.64	8.26 ± 0.68	15.26 ± 0.68
Ascorbic acid, mg	31 ± 6	20 ± 4	19 ± 4	17 ± 4	17 ± 7	14 ± 6	36 ± 9	38 ± 9	77 ± 9	24 ± 4	24 ± 4	64 ± 4	34 ± 6	24 ± 6	64 ± 6

^*∗*^Significant at *p* < 0.0001.

**Table 6:** Adequacy of energy and nutrient intakes of subjects after twelve weeks of intervention (%).

Nutrients	Nonintervention group						Fortified milk group					
	Normal weight (NO)		Underweight (UW)		Severely underweight (SU)		Normal weight (NO)		Underweight (UW)		Severely underweight (SU)	
	% EAR	% RENI	% EAR	% RENI	% EAR	% RENI	% EAR	% RENI	% EAR	% RENI	% EAR	% RENI
Energy	79	79	68	68	70	70	114	114	112	112	110	110
Protein	159	82	132	68	136	70	236	122	245	126	237	122
Iron	185	86	182	85	158	74	462	216	402	187	357	167
Vitamin A	36	29	32	26	29	23	152	122	153	123	148	119
Calcium	40	36	37	34	35	32	228	207	224	204	220	200
Thiamin	170	142	184	153	152	127	276	230	276	230	276	230
Riboflavin	116	97	122	102	98	82	328	273	286	238	258	215
Niacin	142	122	137	117	117	100	312	267	278	238	254	218
Ascorbic acid	80	67	68	57	56	47	309	258	254	212	255	213

*Notes*. EAR: Estimated Average Requirement; RENI: Recommended Energy and Nutrient Intake.

**Table 7:** Subgroup analysis for fortified milk group's psychomotor scores.

		Sum of squares	df	Mean square	*F*	Sig.
PM_PRE	Between groups	266.533	2	133.267	4.040	.023
	Within groups	1880.450	57	32.990		
	Total	2146.983	59			
PM_POST	Between groups	16.533	2	8.267	.175	.840
	Within groups	2696.800	57	47.312		
	Total	2713.333	59			

**Table 8:** Correlation of anthropometric measurements with psychomotor scores.

Anthropometric measurements			Interpretation
Height	Pearson Correlation	−0.11	Very weak negative correlation
	Sig. (2-tailed)	0.40	
	*N*	60	
Weight	Pearson Correlation	0.25	Weak positive correlation
	Sig. (2-tailed)	0.05^*∗*^	
	*N*	60	
BMI	Pearson Correlation	0.18	Very weak positive correlation
	Sig. (2-tailed)	0.16	
	*N*	60	
MUAC	Pearson Correlation	0.28	Weak positive correlation
	Sig. (2-tailed)	0.03^*∗*^	
	*N*	60	

^*∗*^Significant at *p* = 0.05.

**Table 9:** Mean change in different psychomotor skills after twelve weeks of intervention adjusted for covariates (%).

Psychomotor skills	Nonintervention group (*n* = 60)	Fortified milk group (*n* = 60)	*p* value
Putting puzzles together	22.41	68.42	0.0001^*∗*^
Matching outlines in concrete objects	8.48	29.31	0.0115^*∗*^
Comparing and contrasting objects	1.72	13.56	0.0411^*∗*^
Identifying sequential patterns	−24.14	50.00	0.0001^*∗*^
Repeating nonsequential digits	17.24	56.90	0.0001^*∗*^
Sequencing three (3) pictures	−6.55	63.79	0.0001^*∗*^
Identifying functions of body parts	5.17	46.55	0.0001^*∗*^
Recognizing colors	8.93	20.34	0.2995
Identifying shapes	−1.73	10.38	0.2111
Matching shapes	0.00	1.70	0.0759
Matching pairs/go together	−12.07	0.00	0.1577
Identifying objects	18.64	24.14	0.5526
Grouping objects according to function	−10.34	3.45	0.1837
Recalling objects from a given set	1.72	18.64	0.0753
Recalling objects in a picture	15.25	24.14	0.3705
Identifying objects as the same or different	1.72	16.52	0.1399
Matching and disseminating figures	−6.78	−5.26	0.8939
Standing on tiptoe, balancing, and walking	14.29	42.37	0.0041^*∗*^
Hopping on one foot	5.45	27.12	0.0433^*∗*^
Hopping backwards, two (2) feet	0.00	18.64	0.0335^*∗*^
Skipping	5.36	11.86	0.0487^*∗*^
Galloping	10.71	16.95	0.0057^*∗*^
Jumping from stationary position	5.36	22.03	0.0054^*∗*^
Jumping over small objects	5.46	23.73	0.0316^*∗*^
Stringing 1′′ beads with string or shoelace	1.72	13.79	0.1711
Throwing ball	3.45	34.48	0.9996
Catching ball	−1.75	0.00	0.7939
Catching bounced ball	7.02	12.88	0.5747
Kicking rolling ball	−1.79	10.17	0.2150
Balancing on one leg	−3.51	1.70	0.0651
Running smoothly	5.36	13.56	0.1364
Walking down and upstairs with alternating feet	0.00	10.17	0.3852
Walking on 10′′ wide balance beam	−1.72	9.73	0.2041
Climbing playground equipment easily	0.00	12.01	0.3211

^*∗*^Significant at *p* < 0.05.
